# The efficacy of the 3-dimensional vibe-caipirinha-dixon technique in the evaluation of pancreatic steatosis

**DOI:** 10.3906/sag-1909-83

**Published:** 2020-02-13

**Authors:** Ural KOÇ, Gökhan OCAKOĞLU, Oktay ALGIN

**Affiliations:** 1 Section of Radiology, Ankara Sehit Ahmet Ozsoy State Hospital, Ankara Turkey; 2 Department of Biostatistics, School of Medicine, Uludag University, Bursa Turkey; 3 Department of Radiology, School of Medicine, Yıldırım Beyazıt University, Ankara Turkey

**Keywords:** Pancreas, magnetic resonance imaging, chemical shift imaging, MRI scans, steatosis

## Abstract

**Background/aim:**

CAIPIRINHA is a new technique in abdominal imaging. Pancreatic steatosis (PS) is a subject of increasing scientific interest. The aim of this study was to investigate the efficacy of the isotropic 3D-VIBE- CAIPIRINHA -DIXON technique on a new generation 3-tesla MR unit in the evaluation of PS.

**Materials and methods:**

In this retrospective study, the imaging findings of 49 patients with PS and 41 control subjects were examined. The pancreas-to-spleen ratio (PSR), pancreas-to-muscle ratio (PMR), and pancreatic signal intensity index (PSII) were defined as 3 new parameters and these indexes were calculated from the in-phase/out of phase 3D-VIBE- CAIPIRINHA-DIXON images.

**Results:**

The PSR, PMR, and PSII values were significantly different between the patient and control groups (P = 0.001, P = 0.009, P < 0.001, respectively). Statistically significant differences were observed between patient and control groups for ROI measurements of fatty areas on these sequences/images: subtraction (in-out) (P < 0.001), T2W HASTE (P < 0.001), DIXON-fat (P < 0.001), fat-suppressed T1W (P = 0.002), and subtraction (out-in) (P = 0.010).

**Conclusion:**

Evaluation of PS with the 3D-VIBE-CAIPIRINHA-DIXON technique can be made rapidly and effectively.

## 1. Introduction

Pancreatic steatosis (PS) was first defined by Ogilvie in a cadaver study, with determination of relationships between age and metabolic comorbidities such as atherosclerosis and diabetes, and it became a subject in which interest has not diminished [1–4]. It has been shown that fat in the pancreas is related to the severity of pancreatitis, pancreatic cancer, and postoperative pancreatic fistula [5–7]. In recent literature, the clinical results related to PS have been conflicting in publications regarding whether PS is related to pancreatic endocrine or/and exocrine function [8,9]. Tahtacı et al. and Kromrey et al. suggested that pancreatic exocrine impairment was associated with fatty pancreas via magnetic resonance imaging and fecal elastase [8,9]. On the other hand, Miyake et al. reported that fatty pancreas was not associated with pancreatic exocrine impairment but associated with endocrine impairment via computed tomography (CT) [10]. There is no clinical and/or laboratory biomarker of PS, although it can be assessed with radiological modalities. However, PS is often overlooked in radiology practice. Biopsy is the gold standard for exact evaluation of the PS, but because of the location of the pancreas, the complications risk, and sampling bias, radiological methods are preferred for both evaluation and quantification [11]. 

There are some studies in the literature related to the quantification of pancreatic fat content with MRI [11,12]. Techniques such as in phase-opposed phase, dual-echo, or multiecho DIXON method, spectral-spatial excitation, and spectroscopy are used in abdominal MR practice in fat imaging [11–13]. Multiple applications of dual-echo DIXON or multiecho DIXON methods are used for improving fat saturation, quantification, and mapping [11–14]. Quantitative techniques require more echoes for achieving higher values of sensitivity, specificity, and accuracy in detecting steatosis due to the limitations of dual-echo DIXON method [14]. On the other hand, CT is also used for quantification of fat in pancreas [10]. However, there is insufficient use of these methods in routine daily practice to provide effective, objective, or quantitative data. 

More recently, the use of the “controlled aliasing in parallel imaging results in higher acceleration” (CAIPIRINHA) method has been presented, which allows the acquisition of isotropic, high-resolution, 3D abdominal MRI data and reduces imaging time [15–18]. This parallel imaging method is a new technique offering several advantages in abdomen imaging, such as reduced artifacts and shorter acquisition time.

The aim of this study was to investigate the efficacy of the isotropic 3D-VIBE-CAIPIRINHA-DIXON technique on a 3-tesla device in noninvasive evaluation of PS. 

## 2. Materials and methods

Our hospital patient information system (PACS) was scanned to identify all the cases in which 3-tesla noncontrast-material enhanced pancreas MRI was administered during the 2-year period between 2015 and 2017. The images of those patients were retrieved from the picture archiving and communication systems (PACS) and evaluated. Cases diagnosed with PS on these MR examinations (according to radiological MRI report) formed the study group. Cases without PS from the same MR protocol applied during the same period for various reasons (e.g., cyst or mass identification), were accepted as the control group. Cases were excluded if they had a history of malignancy, pancreatic surgery, acute and chronic pancreatitis, autoimmune/IgG4 pancreatitis, pancreatic lesions, blood transfusion, hemochromatosis, chemotherapy, or were diagnosed with iron overload in their records. Approval for the study was granted by the Local Ethics Committee (15.05.2018/24/10) and all procedures were applied in accordance with the Helsinki Declaration. Informed consent was waived because of the retrospective nature of the study.

Two experienced radiologists (O.A. and U.K.) evaluated the images for quality, and 9 cases were excluded from the study due to technical reasons (such as incomplete breath-holding or motion). Radiological MRI reports and images were checked by O.A. for concordance of radiological reports-images and preventing bias in patient selection before evaluation by U.K. As a result, 49 patients (28 female, 21 male; mean age: 69 ± 8 years) with PS and 41 control subjects (18 female, 23 male; mean age: 66 ± 9 years) were included in the study. The final study population was 90 cases (mean age: 66 ± 9 years; range: 43–86 years; 46 female, 44 male).

### 2.1. MRI acquisitions

All cases were examined on a 3-tesla MR unit with a 48-channel system (Magnetom Skyra, Siemens Healthcare, Germany) with 30-channel abdominal coil setup (with 18-channel body and 12-channel from the spine coils). Patients were positioned headfirst and supine with the coils fixed tightly. After the acquisition of localizer, T1 and T2-weighted (W) routine (standard and vendor-optimized noncontrast material enhanced 2D sequences) images for pancreas imaging, pre and postcontrast 3D-VIBE-GRAPPA, and 3D-VIBE-CAIPIRINHA-DIXON sequences were obtained in each case. The 3D-VIBE-CAIPIRINHA-DIXON technique provided in-phase, opposed-phase, fat, and water images, simultaneously. Dual-echo DIXON chemical shift imaging was performed in acquisitions. 3D-VIBE-GRAPPA and 3D-VIBE-CAIPIRINHA-DIXON sequences were performed in random order for all cases. Details of the 3T MRI protocol used are given in Table 1. 

**Table 1:** A 3 Tesla MRI protocol used for the study.

Sequences/parameters	T2-HASTE-FS	T2-HASTE	CAIPIRINHA	T1-VIBE
TR/TE (ms)	1000/95	1000/99	4.21/1.34	4/1.74
Slice thickness (mm)	5	5	1.5	2.2
FOV* (mm2)	380 × 297	400 × 336	450 × 366	400 × 338
Acquisition time (min.s)	1.25	0.57	0.14	0.13
NEX	1	1	1	1
Number of slices	38	26	96	64
Flip angle (°)	160	139	9	18
Imaging plane	Axial	Coronal	Coronal	Coronal
Distance factor (%)	20	10	20	20
PAT factor	2	3	6	3
PAT mode	GRAPPA	GRAPPA	CAIPIRINHA	GRAPPA
Voxel size (mm3)	1.2 × 1.2 × 5	1.2 × 1.3 × 4	1.4 × 1.4 × 1.5	1.3 × 1.3 × 2.2
Fat-saturation	+ (SPAIR)	–	–	+ (Q-fat-sat)
Base resolution	320	320	320	320
Matrix	203 × 320	320 × 320	256 × 192	256 × 192

NEX: number of excitations; FOV: field of view; PAT: parallel acquisition technique; GRAPPA: generalized auto calibrating partially parallel acquisitions.

### 2.2. Image evaluation

All images of the cases were evaluated randomly by an experienced radiologist (U.K.), blinded to clinical history. The findings mentioned below were analyzed, and related grades/scores/measurements were recorded in the relevant column of the study table. Some quantitative ratios (e.g., pancreatic signal intensity index, pancreas to spleen/muscle ratios) were determined by the authors and these ratios were calculated and recorded. 

### 2.3. Qualitative analyses

PS was visually analyzed on the T1W fat-saturated (FS) and T2W-HASTE images with a 3-point scale (grade 1: 0%–33%, grade 2: 34%–66%, grade 3: 67%–100%). Pancreatic atrophy was assessed visually using a 4-point scale (grade 0: none; grade 1: minimal; grade 2: moderate; grade 3: prominent) taking the data of all the sequences into consideration. Pancreas atrophy criteria were fat accumulation in pancreas and a decrease in pancreatic parenchyma size in visual radiological evaluation. The localization of PS was evaluated using a 5-point scale (grade 0: none; grade 1: diffuse; grade 2: head-neck; grade 3: body; grade 4: tail) taking all the sequences into consideration.

Fatty areas were assessed with a 3-point scale (hypo-intense: 0; iso-intense: 1; and hyper-intense: 2) for all sequences. If fatty areas were observed as hyperintense on in-phase images and hypointense on out-phase images, this situation was accepted as suppression phenomenon (chemical shift effect on DIXON images). This suppression on in-phase and out-phase images of fatty regions was assessed with a 4-point scale (absent: 0; minimal: 1; moderate: 2; and prominent: 3).

The 3D-VIBE-CAIPIRINHA-DIXON data set assessment was made visually by evaluating DIXON fat, water, in-phase, and out-phase images together.

### 2.4. Quantitative analyses

**Figure 1:**
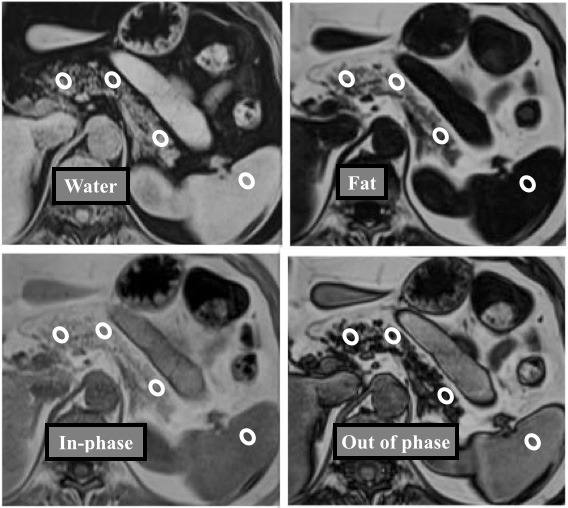
The measurements were made in the head-neck, body, and tail sections of the pancreas and spleen from a region of interest (ROI) of approximately 1 cm2 on 3D-VIBE-CAIPIRINHA-DIXON data set (water, fat, in phase, out phase subgroup images).

For the determination of signal/noise ratios (SNRs) on DIXON-subgroup and subtraction images, an approximately 1 cm2 region of interest (ROI) was drawn on the fatty area of the pancreas, not to go beyond the fatty area (not including structures such as large vessels and the main pancreatic canal) and the signal intensity (SI) of the pancreatic tissue was determined (Figure 1). On the same slice, a 1 cm2 ROI was placed on the noise area outside the body, and the SNR was calculated. The contrast to noise ratio (CNR) was calculated as the SNR measurement of the target area value divided by the spleen SNR value. 

The fat fraction % (FF%) value was calculated as a percentage by dividing the SI value of the fatty area on the fat images by the total SI value of the fatty areas on the fat and water images (FF%) = (F/F + W)%. The adrenal to spleen and adrenal signal intensity index formula previously described by Savcı et al. for the evaluation of adrenal masses were adapted to the pancreas [19]. These ratios were calculated as follows:

Pancreas to spleen ratio (PSR) = ([SI pancreas/SI spleen] on out-phase images / [SI pancreas /SI spleen] on in-phase images – 1) × 100

Pancreas to muscle ratio (PMR) = ([SI pancreas/SI muscle] on out-phase images / [SI pancreas /SI muscle] on in-phase images – 1) × 100

Pancreatic signal intensity index (PSII) = (SI pancreas in phase – SI pancreas out phase) / (SI pancreas in phase) × 100

The quantitative measurements were obtained by a single researcher (U.K.) blinded to the clinical data.

### 2.5. Statistical analyses

The conformity of variables to normal distribution was assessed using the Shapiro–Wilk test. Variables were reported as median (minimum:maximum) values. According to the normality test result, the Mann–Whitney U test and Independent samples t test were used for between-group comparisons. Categorical variables were compared using the Chi-square test, Fisher–Freeman–Halton test. The relationships between continuous and discrete variables were examined using correlation analysis and the Pearson/Spearman correlation coefficient was calculated. ROC curve analysis was applied to determine the FF% threshold values of each PS grade. The agreement between sequences was determined using Cohen’s kappa coefficient (κ). SPSS version 21 software (IBM Corp., Armonk, NY, USA) was used when performing statistical analyses and the level of significance was set at α = 0.05. 

## 3. Results

According to age and gender distribution, there was no statistically significant difference between the groups (P > 0.05).

PS of varying degrees was observed in the majority of cases in the patient group, and there was generally minimal PS in control group cases (Table 2). A statistically significant difference was determined between the groups in respect to the presence of PS (P < 0.001, Table 2).

**Table 2:** Comparison of morphological characteristics of fatty-areas between patient and control groups.

Pancreatic steatosis on HASTE images	Patient group	Controls	P value
0%–33% (grade 1)	15 (27.8%)	39 (95.1%)	<0.001a
34%–66% (grade 2)	22 (44.9%)	2 (4.9%)	
67%–100% (grade 3)	12 (24.5%)	0	
Pancreatic steatosis on T1WFS images	Patient group	Controls	P value
0%–33% (grade 1)	30 (61.2%)	41 (100%)	<0.001a
34%–66% (grade 2)	16 (32.7%)	0	
67%–100% (grade 3)	3 (6.1%)	0	
3D-VIBE-CAIPIRINHA-DIXON data set assessment	Patient group	Controls	P value
0%–33% (grade 1)	24 (49%)	41 (100%)	<0.001a
34%–66% (grade 2)	19 (38.8%)	0	
67%–100% (grade 3)	6 (12.2%)	0	
Appearance of fatty-areas on fat images	Patient group	Controls	P value
Isointense (score 1)	5 (10.2%)	25 (61%)	<0.001a
Hyperintense (score 2)	44 (89.8%)	16 (39%)	
Suppression on out-phase images	Patient group	Controls	P value
Absent (score 0)	6 (12.2%)	26 (63.4%)	<0.001b
Minimal (score 1)	17 (34.7%)	11 (26.8%)	
Moderate (score 2)	18 (36.7%)	4 (9.8%)	
Prominent (score 3)	8 (16.3%)	0	
Appearance of fatty-areas on out-phase images	Patient group	Controls	P value
Hypointense (score 0)	43 (87.8%)	15 (36.6%)	<0.001b
Isointense (score 1)	0	1 (2.4%)	
Hyperintense (score 2)	6 (12.2%)	25 (61%)	

aPearson chi-square test; bFisher–Freeman–Halton test.

In general, the pancreatic fatty areas in the patient group were seen as hypointense on out-phase images and hyperintense on fat images; in the control group cases, the fatty areas showed a heterogeneous signal intensity pattern on out-phase images (Table 2). The difference between the two groups in this respect was statistically significant (P < 0.001, Table 2). In 88% of cases in the patient group, suppression was observed on the in-phase and out-phase images of the PS areas (Table 2).

Distribution of pancreatic atrophy grades and fatty areas appearances of the patient group are given in Table 3. A statistically significant difference was determined between the patient and control groups in respect of the distribution of fatty replacement sites (P < 0.001, Table 3). 

**Table 3:** Pancreatic atrophy and fatty replacement areas comparisons of the patient and control groups. Data were given as number and percentage (%).

Pancreatic atrophy	Patient group	Controls	P value
None	24 (49%)	32 (78%)	0.001b
Minimal	13 (26.5%)	9 (22%)	
Moderate	10 (20.4%)	0	
Prominent	2 (4.1%)	0	
Fatty replacement sites	Patient group	Controls	P value
Diffuse	45 (91.84%)	9 (21.95%)	<0.001b
Only head-neck	0	0	
Only body	3 (6.12%)	4 (9.76%)	
Only tail	1 (2.04%)	3 (7.32%)	

bFisher–Freeman–Halton test.

Pancreatic atrophy was determined in 25 (51%) cases of the patient group and in 9 (22%) cases of the control group (P = 0.001). In the control group, in subjects with pancreatic atrophy, it was determined at a mild level (Table 3). The fatty replacement pattern was usually diffuse in cases with minimal PS (Table 3). 

In respect of PSR, PMR, and PSII variables, there were statistically significant differences between the groups (P = 0.001, P = 0.009, P < 0.001, Table 4). The ROI measurements of the fatty areas on subtraction in-out of phase, subtraction out-in phase, and fat images were statistically significantly different between the patient and control groups (P < 0.001, P = 0.01, P < 0.001, Table 4). 

**Table 4:** Quantitative study parameters of the patient and control groups.

Parameters	Patients (n = 49)	Controls (n = 41)	P value
	Median (minimum : maximum)	Median (minimum : maximum)	
PSR	–45.77 (–84.38 : 177.86)	–21.08(–66.85 : 15.63)	0.001c
PMR	–25.49(–76.11 : 175.33)	–2.87(–56.13 : 128.79)	0.009c
PSII	49.40(–167.86 : 75.61)	22.08 (1.12 : 69.94)	<0.001c
Fatty areas SI on subtraction out-in	3 (0 : 12)	4 (0 : 45)	0.010c
Fatty areas SI on subtraction in-out	172 (32 : 344)	82 (19 : 272)	<0.001d
Fatty areas SI on fat images	177.81 (23.44 : 327.83)	55.70 (13.95 : 99.08)	<0.001c
CNR of fatty areas on fat images	8.11 (0.52 : 22.33)	2.06 (0.42 : 6.25)	<0.001c
SNR of fatty areas on fat images	19.04 (0.36 : 54.53)	9.61 (1.62 : 38.85)	0.001c
Fat fraction via SI	57.34 (8.50 : 83.82)	17.83 (6.45 : 31.96)	<0.001c
Fat fraction via SNR	38.83 (2.11 : 73.41)	10.23 (3.02 : 43.29)	<0.001c
Fat fraction via CNR	91.64 (31.14 : 98.67)	65.73 (31.28 : 85.83)	<0.001c
Spleen intensity on fat images	22.62 (12.54 : 72.73)	24.24 (11.74 : 95.95)	0.916c
Spleen signal intensity index	6.51 (–99.57 : 35.10)	5.52 (–64.49 : 31.03)	0.613c

cMann–Whitney U test; dIndependent samples t test.

For ROI measurements of fatty areas, there were statistically significant positive correlations between out phase images and subtraction out-in, water images, or PMR/PSR ratios for all patients (Table 5). In addition, there were inverse or negative relationships between fatty areas on out-phase images and subtraction in-out/fat images, or PSII values (Table 5). Also, there are many negative or positive correlations for many study parameters between in-phase and out-phase data for fatty-region ROI measurements (Table 5).

**Table 5:** Relationships between ROI values of fatty areas and other study parameters.

Parameters	Fatty areas onin-phase images		Fatty areas onout-phase images		r	P	r	P
	Fatty areas on subtraction out-in	–0.04e	0.693	0.26e	0.013
Fatty areas on subtraction in-out	0.20f	0.055	–0.48f	<0.001
ROI values of fatty areas on fat images	0.35e	0.002	–0.51e	<0.001
ROI values of fatty areas on water images	0.26f	0.024	0.72f	<0.001
PSR	0.14e	0.199	0.62e	<0.001
PMR	0.08e	0.433	0.45e	<0.001
PSII	–0.06e	0.563	–0.60e	<0.001
CNR of fatty areas on fat images	0.34e	0.003	–0.37e	0.001
SNR of fatty areas on fat images	0.11e	0.365	–0.32e	0.005
Fat fraction via signal intensity	0.16e	0.178	–0.59e	<0.001
Fat fraction via SNR	0.10e	0.414	–0.57e	<0.001
Fat fraction via CNR	0.29e	0.013	–0.47e	<0.001

eSpearman correlation coefficient; fPearson correlation coefficient.

**Figure 2:**
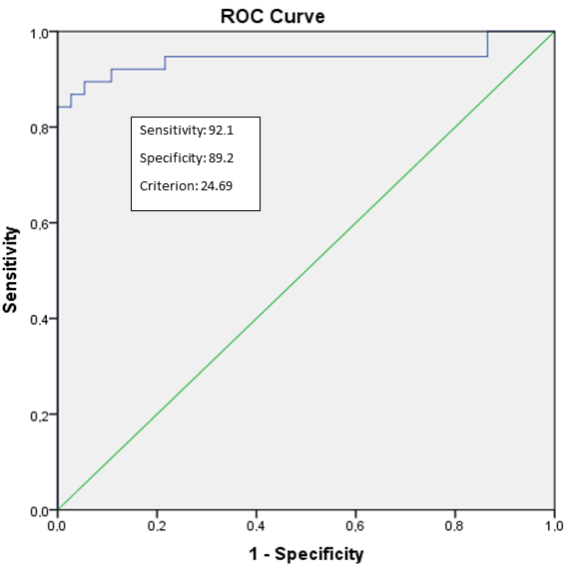
Receiver-operator characteristic (ROC) curves for the diagnosis of PS between patient and control group using the 3D-VIBE-CAIPIRINHA-DIXON data set. The area under the curve (AUC) for 3D-VIBE-CAIPIRINHA-DIXON data set is 0.94 (95% CI: 0.88–1.00) with P < 0.001.

**Figure 3:**
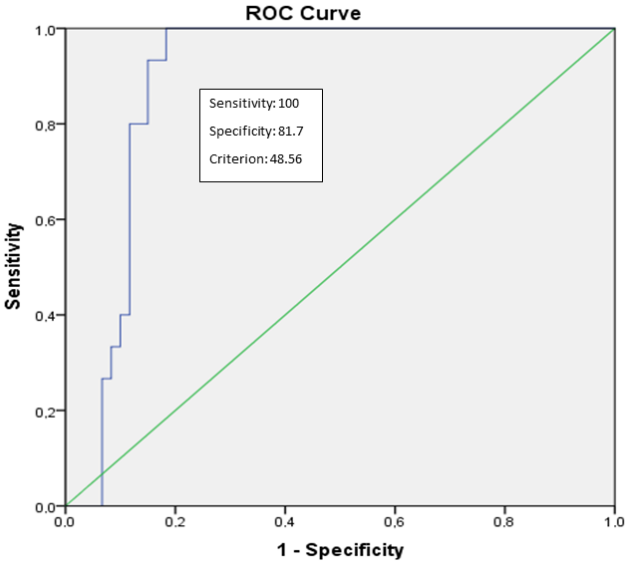
Receiver-operator characteristic (ROC) curves for the diagnosis of grade 2 steatosis using the 3D-VIBE-CAIPIRINHA-DIXON data set. The area under the curve (AUC) for 3D-VIBE-CAIPIRINHA-DIXON data set is 0.89 (95% CI: 0.82–0.97) with P < 0.001.

**Figure 4:**
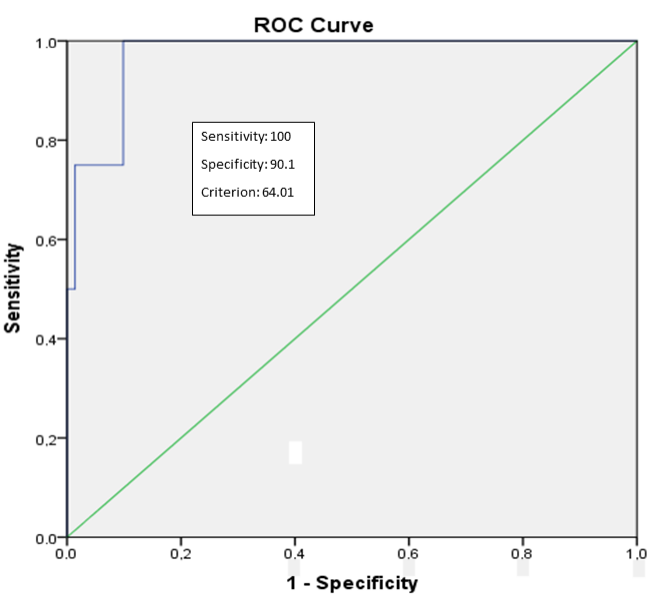
Receiver-operator characteristic (ROC) curves for the diagnosis of grade 3 steatosis using 3D-VIBE-CAIPIRINHA-DIXON data set. The area under the curve (AUC) for 3D-VIBE-CAIPIRINHA-DIXON data set is 0.97 (95% CI: 0.92–1.00) with P = 0.002.

FF% cut-off value for the diagnosis of PS with given sensitivities and specificities was determined to be 25% (sensitivity: 92.1%; specificity: 89.2%; AUC: 0.94; P < 0.001; 95% CI 0.88–1.00) (Figure 2). FF% value > 48.6% was determined as the cut-off value for the diagnosis of grade 2 PS (sensitivity: 100%; specificity: 82%; AUC: 0.89; P < 0.001; 95% CI 0.82–0.97) (Figure 3). ROC analyses showed that FF% value > 64% was the diagnostic cut-off value for grade 3 PS (sensitivity: 100%; specificity: 90%; AUC: 0.97; P = 0.002; 95% CI 0.92–1.00) (Figure 4).

There was moderate agreement between 3D-VIBE-CAIPIRINHA-DIXON data and HASTE or T1WFS (0.623, 0.687, respectively; P < 0.001). Also, there was minimal agreement between HASTE and T1WFS (0.392; P < 0.001) images.

## 4. Discussion

The point of motivation for this study was to evaluate the efficacy of the 3D-VIBE-CAIPIRINHA-DIXON technique in the cases of pancreatic steatosis. The main reasons for this preference were the consideration of homogenous fat-suppression with this technique and fewer motion artifacts due to a faster acquisition, leading to improved delineation of the pancreas on 3D subgroup images (especially fat, water, and subtraction images) as highlighted in the literature. There are very few studies in the literature related to CAIPIRINHA and MR evaluation of PS [20–23]. The CAIPIRINHA technique is relatively new and has started to be used in various clinical protocols in abdominal imaging [22,23]. The use of the CAIPIRINHA together with the DIXON technique, which is a chemical shift-based fat saturation technique, has been reported in literature as an element increasing image quality in isotropic-3D abdominal imaging [18]. CAIPIRINHA enables the acquisition of satisfactory image quality during the time of holding a single breath, thereby overcoming the long scanning time, which is the most significant disadvantage of the DIXON technique [18]. In a recent study, it was reported that CAIPIRINHA-DIXON provided high temporal resolution without loss of spatial resolution [22]. Furthermore, the use of CAIPIRINHA together with the DIXON technique allows more homogenous fat suppression on isotropic 3D imaging. Consequently, 3D-VIBE-CAIPIRINHA-DIXON increases contrast between the pancreas and surrounding peripancreatic tissues. The pancreas can be evaluated in multiple planes and at high CNR with a single acquisition by 3D-VIBE-CAIPIRINHA-DIXON [18]. 

When the clinical reflection of PS is examined, there are publications that report endocrine and exocrine insufficiency or disorders, increased severity of pancreatitis, development of pancreatic cancer, and postoperative pancreatic fistula on the basis of fatty pancreas [5–7,11,24–26]. CT is one of the tools for quantification of fat in the pancreas [10]. Pancreas to spleen density ratio (P/S) via CT is the current indicator of interest for the prediction of postoperative pancreatic fistula development before pancreatoduodenectomy procedure and endocrine and exocrine insufficiency or disorders [10,27,28]. Fukie et al. reported that MR-based pancreatic fat quantification was superior to CT-based quantification for estimating the probability of pancreatic cancer [29].

Acquisition time of conventional out-phase/in-phase imaging without acceleration is approximately 60 s. In this study, PS was evaluated on 3D-VIBE-CAIPIRINHA-DIXON images obtained within a single breath (acquisition time of 14 s). The SNR and CNR ratios of the fatty areas were calculated, and to be able to evaluate PS quantitatively, the new parameters of PSR, PMR, and PSII were defined. For fatty areas, there was a significant negative correlation between SI values of 3D-VIBE-CAIPIRINHA-DIXON out of phase images and SI values of 3D-VIBE-CAIPIRINHA-DIXON fat images, PSII ratio, and FF%. This negative correlation can be considered to be due to loss of signal seen in out of phase imaging in the areas of steatosis. In addition, in the current study, a significant positive correlation was determined between PSR/PMR values and signal intensities of fatty areas on subtraction (out-in phase) images. According to the above-mentioned results, PSR, PMR, and/or PSII ratios can be useful for quick and quantitative assessment and follow-up of PS and the pancreas. In the preoperative planning or follow-up of PS or pancreatic atrophy, the 3D-VIBE-CAIPIRINHA-DIXON and the parameters derived using this technique can be used within a short time and free of radiation.

Consistent with previous findings in literature, statistically significant differences were determined between the patient and control groups with respect to many MR parameters for PS evaluation [23,30]. Ghasabeh et al. reported that dual-echo and multiecho DIXON methods revealed moderate and good correlations with each other and magnetic resonance spectroscopy (MRS) related to fat measurements in liver and pancreas [14]. In our study, we used a dual-echo DIXON method for quantification of fat with parallel imaging technique, CAIPIRINHA. For the detection of pancreatic fat, FF% can be calculated using signal intensities (SI) from out-phase and in-phase images via chemical shift techniques [21]. The results of this study showed a good correlation between the FF% values calculated using DIXON fat and water subgroup images and the grading on the other images. In the FF% analyses, if the cut-off value was selected as 24.69%, FF% sensitivity was 91.2% and specificity was 80.5% (based on final decisions by 3D-VIBE-CAIPIRINHA-DIXON data set). In general, as FF% values increase, the sensitivity decreases and the specificity increases (or vice versa).

The parameter commonly used in the literature [19] for fat detection, such as signal intensity ROI values on out phase images and the findings in the routine imaging sequences, show a good correlation with the PSII parameter, which has been defined in this study for the first time in literature and was derived from the image data set obtained using this technique. This parameter can also be used as a reliable indicator for fat measurements.

T2W fast-spin echo, HASTE, and/or T1W sequences with and without fat-saturation are generally used in routine pancreatic imaging [31]. Homogenous fat suppression for these techniques or pancreatic imaging are needed. In general, conventional fat-saturation techniques provide heterogeneous fat, although the long echo train length in the T2W HASTE sequence reduces spatial resolution, SNR, and CNR parameters [32–34]. This leads to limitations in the evaluation [33–34], and these limitations can be overcome with the DIXON technique [18].

The results of this study demonstrated differences between the data obtained from the routine sequences and the 3D-VIBE-CAIPIRINHA-DIXON data set. The study results showed that the nonfat saturated T2W HASTE technique caused overestimation according to 3D-VIBE-CAIPIRINHA-DIXON in PS evaluation, whereas the T1W-FS resulted in underestimation according to 3D-VIBE-CAIPIRINHA-DIXON. When the agreement between the sequences was examined, there was moderate agreement between the 3D-VIBE-CAIPIRINHA-DIXON data set assessment and HASTE and T1WFS, and minimal agreement between HASTE and T1WFS. These findings reveal the nonconformity of evaluating a single sequence when evaluating the PS. 3D-VIBE-CAIPIRINHA-DIXON increases the confidence of the evaluator as well as reducing acquisition time. 3D-VIBE-CAIPIRINHA-DIXON allows us a more robust pancreatic assessment with the benefits of DIXON, which allows homogenous fat suppression, and CAIPIRINHA, which makes a positive contribution to CNR, and provides 3D isotropic imaging.

In an MRI-based study by Wong et al., PS prevalence was determined as 16% in healthy subjects and PS seen at up to 10.4% was accepted as a normal condition, thereby establishing a cutoff value of 10.4% for PS [13]. In the current study, the vast majority of the control cases were determined as grade 1 (0%–33%), similar to the literature. A previous study determined minimal (grade 1) PS in cases determined with grade 1 nonalcoholic fatty liver disease [20]. That minimal PS was observed in the majority of our control group and suppression was observed at a rate of 37% (15/41) on the out of phase images. This can be explained by the mean age of the control group being relatively high and that therefore a certain level of fat content would be found in the normal pancreas. A positive correlation between age and PS is known to exit [2,3]. However, in the patient group, grade 1 PS was determined at 49% and grades 2 and 3 were determined at 51%. Minimal, moderate, and advanced suppression was determined on the out-phase images in 87.8% of the patient group, which was statistically significantly higher compared to the control group (P < 0.001).

When evaluating the distribution of fatty areas, most of the current cases were seen to have diffuse type PS. This finding was consistent with the literature data [20,21]. Another finding of the study was the determination of pancreatic atrophy at 22% (9/41) in the control group, and at 51% (25/49) in the patient group (P = 0.001). These results are evidence of a relationship between PS and pancreatic atrophy. 

Savcı et al. argued that the suppression effect on the in-phase and out-phase images is a sign of intracellular fat accumulation [19]. Our study revealed that suppression was observed in fatty areas on the in-phase and out-phase images in 88% of cases with PS. This result suggests that the cause of PS is mostly due to intracellular fat accumulation. According to our knowledge, this information is not yet available in the radiology or imaging literature.

There were some limitations to this study. First, no comparison was made of the MR findings with the histopathological evaluation, which is the gold standard. On the other hand, magnetic resonance spectroscopy (MRS) shows a good correlation with mDIXON for assessment of hepatic and pancreatic fat fraction [29]. MRS has been known as the second most accepted standard approach to quantification of fat noninvasively but is challenging due to the size of the pancreas, artefacts such as motion, several technical limitations such as software requirements, and amount of time required [14]. Proton density fat fraction (PDFF) is a commonly used technique for direct quantification of fat in abdominal imaging [30]. The retrospective nature of the study and the lack of PDFF application when images were obtained led to the ROI measurements of pancreatic signal intensity fat fraction. The reliability of multiecho, dual-echo DIXON ROI measurements of pancreatic signal intensity fat fraction can be tested via PDFF technique or MRS. Inter/intraobserver agreement was not evaluated, and the study population was relatively low. There is a need for further studies with larger patient series, taking these points into consideration.

In conclusion, in our study, 3D-VIBE-CAIPIRINHA-DIXON was preferred for accurate and fast assessment of PS. PSII, a newly described parameter, can be used as a quantitative indicator in the evaluation and follow-up of PS. Also, the cause of PS is mostly due to intracellular fat accumulation. This study can be considered of value as a preliminary study that can be of guidance for further clinical and radiological studies.
